# Supplementary material for: A smoothed particle hydrodynamics study of the collapse for a cylindrical cavity
Source: PLoS One. 2020 Sep 29;15(9):e0239830. doi: 10.1371/journal.pone.0239830 (PMC7523992; doi:10.1371/journal.pone.0239830)
Supplement: Attachment — Submitted filename: Response to Reviewers.docx [file pone.0239830.s002.docx]

- **Editor**

  - Since this study involves the use of computational software, it would need to be deposited as an open access software. I personally use GitHub, but please consult the PLoS One requirements on software for guidance.

Answer:

Open access software was used for the simulation and data post process. All relevant information was added in the revised manuscript: see new section *Software for simulation, visualisation and post-process* in *Model* page 7, lines 204-207.

  - The energy balance equation includes both viscous dissipation and thermal diffusion. It is not clear if these effects are being separated out when the authors talk about investigating "heat diffusion". The wording "heat diffusion" is awkward. Heat is the transfer of energy by virtue of temperature differences. We often use the term thermal diffusion when referring to Fourier's Law since it is expresses heat as being proportional to temperature differences. It is not clear how viscous dissipation is separated or lumped into the energy analysis.

Answer:

In Eq. 9, the first term of the right hand side represents the sum of the reversible rate of internal energy increase by compression and the irreversible rate of internal energy increase by viscous dissipation. The second term is the particle form of the internal energy change by heat conduction following the Fourier’s law. In the adiabatic collapse simulation only the first term of Eq. 9 is accounted for. In the heat diffusive collapse, both term of Eq. 9 are used in the simulation.

In the revised manuscript, this was added at page 4 lines 100-104 and page 11 lines 324-326.

 - There is a typo in Eq. 8, the pressure tensor is missing.

Answer:

The typo has been corrected. Besides, Eq. 8 has been re-written to better mirror its particle form counter part.

Additional note for the editor:

During the revision of this manuscript Joshi et al published another work where they explain better how their model has been developed. For this page 2 lines 56-59 have been re-written and a new reference has been added.

- **Reviewer 1**
On the whole, I believe that this manuscript is suitable for publication. However, I have some remarks which authors should take into consideration:

- Rayleigh collapse is usually associated with a spherical cavity. Thus, it seems that it might be more appropriate to change the title of the manuscript to ‘A Smoothed Particle Hydrodynamics study of the collapse for a cylindrical cavity’"

Answer:

The title was changed as suggested.

- It may be useful to compare the results of this manuscript with the results of the works where heat transfer was taken into account in Rayleigh-Plesset like models (see, e.g. A. Prosperetti, J. Fluid Mech. 222 (1991) 587–616., Stricker L., Prosperetti A., Lohse D. J. Acc. Soc. Am. 130 (2011) 3243-3251.)

Answer:

We are aware of these publications. However, these works refer to oscillating bubbles, whereas our study focuses on the full bubble collapse. We prefer not to include them in the revised manuscript to avoid the risk of confusing the reader about the main focus of our paper.

- For the verification of the numerical approach authors may also use the experimental and theoretical results from works D. Obreschkow, M. Bruderer, M. Farhat, Phys. Rev. E. 85 (2012) 066303, D. Obreschkow, M. Tinguely, N. Dorsaz, P. Kobel, A. de Bosset, M. Farhat, Exp. Fluids. 54 (2013) 1503.

Answer:

These works are now quoted in the revised manuscript at page 10 lines 288-289 and page 11 lines 331-333.

- Reference 15 has two authors. Thus, it should be cited in the text of the manuscript either by its number or by the last names of both authors. I would also like to draw attention of the authors to another work on analytical studying of bubbles dynamics (N.A. Kudryashov, D.I. Sinelshchikov, Phys. Lett. A 379 (2015) 798–802.), where surface tension was taken into account..

Answer:

These works are now quoted in the revised manuscript at page 2 lines 29-32.
